# Combined Negative- and Positive-Pressure Ventilation for the Treatment of ARDS

**DOI:** 10.1155/2015/714902

**Published:** 2015-07-28

**Authors:** Konstantinos Raymondos, Jörg Ahrens, Ulrich Molitoris

**Affiliations:** ^1^Anaesthesiology and Intensive Care Medicine, Hannover Medical School, 30625 Hannover, Germany; ^2^Anaesthesiology and Intensive Care Medicine, Klinikum Links der Weser, 28277 Bremen, Germany; ^3^Cardiothoracic, Transplantation and Vascular Surgery, Hannover Medical School, 30625 Hannover, Germany

## Abstract

*Objective*. Tracheal intubation and positive-pressure ventilation as the current standard of care for the adult respiratory distress syndrome (ARDS) seem to have reached their limit in terms of a further relevant reduction of the still very high mortality. *Case Presentation*. A 75-year-old male patient developed ARDS after abscess drainage with deteriorating oxygenation, despite positive end-expiratory pressure (PEEP) values above 15 cm H_2_O. We applied external negative-pressure ventilation with a chamber respirator using −33 cm H_2_O at inspiration and −15 cm H_2_O at expiration, combined with conventional pressure support using a PEEP of about 8 cm H_2_O and a pressure support of 4–12 cm H_2_O. Alveolar infiltrates disappeared rapidly and PaO_2_/FiO_2_ values surpassed 300 mmHg after the first application and 500 mmHg after the second. Negative-pressure ventilation was used for 6–18 hours/day over five days. Now, 13 years later, the patient is still alive and has a good quality of life. *Conclusion*. Using this or similar concepts, not only in intubated patients but also as a noninvasive approach in patients with ARDS, offers new options that may genuinely differ from the present therapeutic approaches and may, therefore, have the potential to decrease the present high mortality from ARDS.

## 1. Introduction

Continuous positive-pressure ventilation (CPPV) still represents the standard method of treating the adult respiratory distress syndrome (ARDS), despite the fact that it aggravates both lung injury and multisystem organ failure [1], with mortality from ARDS still as high as 50% [2, 3]. However, there are alternatives for the symptomatic treatment of ARDS. Decades ago, modified Emerson tank respirators were successfully used to treat ARDS with continuous negative pressure [4–6]. These patients had pneumonia and were neither intubated nor ventilated but breathed spontaneously surrounded by negative pressure [4–6]. According to observations of lung injury, it is not only spontaneous breathing under negative pressure that differs substantially from CPPV: this is certainly also the case for ventilation with negative pressure. Continuous external negative-pressure ventilation (CENPV) improves oxygenation under more physiological conditions with lower transpulmonary, airway, and intra-abdominal pressures than with CPPV [7, 8].

Apart from these physiological studies, there are no reports on clinical experience with CENPV in ARDS or the combined use of negative- and positive-pressure ventilation.

## 2. Clinical Case

**Figure 1:**
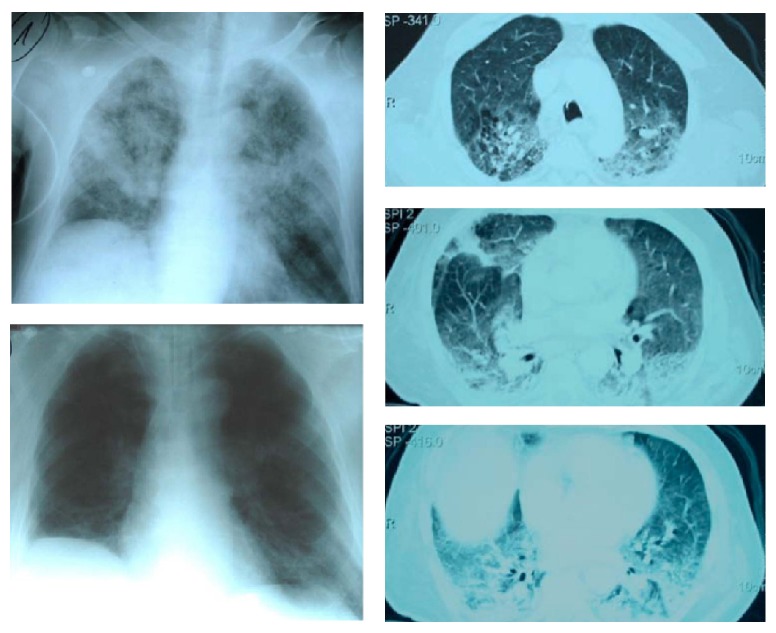
Chest X-rays and CT scan. Patchy bilateral infiltrates can be seen in the first chest X-ray made immediately postoperatively (top left). After three days of noninvasive ventilation and two days of invasive ventilation, the CT scan of the lungs was performed at a PEEP of 15 cm H_2_O (right) showing bilateral dorsal infiltrates reaching from cranial (top right) to caudal lung regions (down right). The chest X-ray made after CENPV during the following day shows impressive regression of the infiltrates and pleural effusions (bottom left), corresponding to improved oxygenation.

A 75-year-old male patient suffered from a peritonsillar abscess, which extended down to the hypopharynx. After anesthesia induction, the patient was intubated without any problems, and tonsillectomy and abscess drainage were performed. Following extubation, the peripheral oxygen saturation in the anesthesia recovery room decreased to 85% despite oxygen administration. Coarse bubbling and moist rales were auscultated over both lungs. Administration of intravenous furosemide administrations had no effect, and the initial chest X-ray revealed patchy bilateral infiltrates but without enlarged heart (Figure 1). A cardiac etiology for the pulmonary edema was ruled out, also clinically, as a physical examination failed to reveal heart murmurs, jugular venous distension, or peripheral edema, and there was no sign of hemodynamic instability. The patient was ventilated noninvasively via a facemask with positive end-expiratory pressure (PEEP) of at least 5 cm H_2_O and, from the second day, with a FiO_2_ of 0.5. On the third day, respiratory insufficiency deteriorated and the patient was intubated. Two days later the patient was tracheostomized; CT scans of the lungs were performed at a PEEP level of 15 cm H_2_O and showed bilateral patchy infiltrates and dorsal consolidation extending from cranial to caudal lung regions (Figure 1).

**Figure 2:**
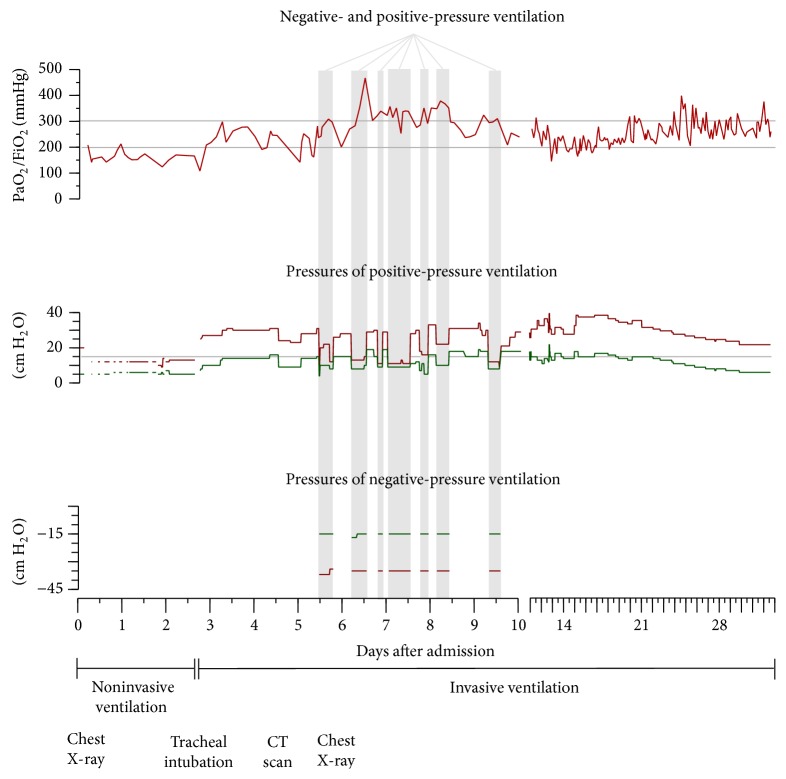
Course of gas exchange and ventilatory pressures. After three days of noninvasive positive-pressure ventilation, the patient was intubated and ventilated in a pressure-controlled mode. Three days later, negative-pressure ventilation was commenced in combination with conventional pressure support (gray columns). In both ventilatory modes, inspiratory pressures are shown as red and expiratory pressures as green lines. Ventilatory pressures represent inspiratory plateau pressures and positive end-expiratory pressures (PEEP) during conventional positive-pressure ventilation and maximum or minimum chamber pressures during negative-pressure ventilation.

**Figure 3:**
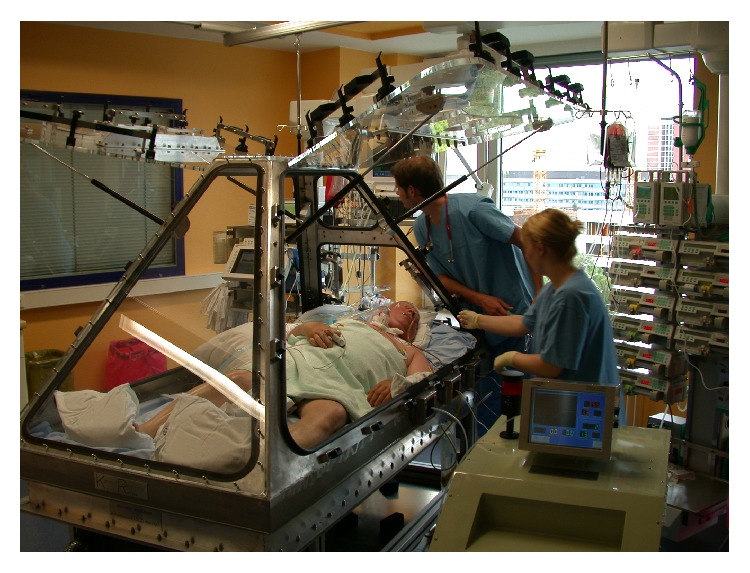
Patient with ARDS in the chamber respirator. CENPV has been discontinued and the chamber opened to enable better access for nursing procedures. The PEEP was elevated before opening the chamber to compensate for discontinuation of end-expiratory negative chamber pressure, and the patient is breathing spontaneously while receiving pressure support from the conventional ventilator. The separate pump unit (bottom right) was manufactured by Coppa S.r.l. (Biella, Italy) and was usually integrated into a tank respirator.

During the following day, the PaO_2_/FiO_2_ ratio again decreased below 200 mmHg, despite high PEEP values of not less than 15 cm H_2_O (Figure 2) that were not sufficient to maintain lung volumes; even recruitment maneuvers using airway pressures up to 85 cm H_2_O were unable to substantially improve oxygenation. In this situation, six days after the development of ARDS and three days after invasive CPPV, we decided to apply CENPV with the chamber respirator (Figure 3).

This device was constructed drawing on our clinical experience with a tank respirator that was built in our hospital in the early 1980s [7]. Gull-wing doors at both sides and one at the head enabled rapid access to the intubated patient who was placed completely inside the chamber (Figure 3). Three air-sealing outlets at each side and two at the top enabled simple and rapid introduction of medical support devices; moreover, during their introduction (or when the doors were opened/closed), there was no need to disconnect any lines or connections.

After receiving permission from the patient's next of kin we started CENPV, applying chamber pressures of −33 cm H_2_O at inspiration and −15 cm H_2_O at expiration. We combined CENPV with conventional PEEP of about 8 cm H_2_O and pressure support of 4–12 cm H_2_O (Figure 2), applying tidal volumes of 6–8 mL/kg predicted bodyweight. For pressure support, we used the BIPAP assist mode of a conventional intensive care respirator (Evita 4, Dräger, Lübeck, Germany).

During the first application of the chamber ventilator (that lasted for 7 hours), the PaO_2_/FiO_2_ value increased from 236 to more than 300 mmHg (Figure 2), and alveolar infiltrates showed an impressive decrease. However, it was only during the second application time (after eight hours of CENPV) that the PaO_2_/FiO_2_ value increased to more than 500 mmHg (Figure 2), indicating significant recruitment of lung volume without the use of any recruitment maneuvers. Before opening the chamber respirator again, the PEEP level was elevated by at least 8 cm H_2_O to compensate for the discontinuation of the end-expiratory negative chamber pressure (Figure 2).

During these first two applications of the chamber respirator, the patient was deeply sedated and CENPV was applied with a fixed setting without triggering the negative-pressure pump. During subsequent applications of CENPV, the patient's spontaneous breathing efforts triggered the external negative-pressure pump unit (Coppa, Biella, Italy) of the chamber ventilator via a thermistor that was fixed at the tracheal cannula. The resulting breaths triggered the pressure support function of the conventional respirator.

After applying CENPV for 5 days for 6–18 h/day, we decided to wean the tracheostomized patient from the ventilator and increased the PEEP to 19 cm H_2_O before opening the chamber ventilator. Following cessation of CENPV, the patient breathed spontaneously at this high PEEP level with pressure support of only 4–8 cm H_2_O (Figure 3). Most remarkably, he breathed slowly and very effectively, making almost sole use of his diaphragm and only very marginal use of his accessory respiratory muscles. Subsequently, higher levels of pressure support became necessary and oxygenation deteriorated again, with PaO_2_/FiO_2_ values mainly between 200 and 300 mmHg (Figure 2). Three weeks after CENPV, the patient was successfully extubated and discharged to the normal ward.

Now, 13 years later, the patient is still alive and has a good quality of life, travelling extensively with his wife and enjoying his grandchildren, and one of his favorite and frequent activities is cycling.

## 3. Discussion

This patient probably developed ARDS due to the focus of infection that spread following abscess drainage; however, the definitive reason for the immediate postoperative development of lung injury remains unknown. Despite high pressure values, oxygenation could not be maintained using PEEP, and recruitment maneuvers proved unable to recruit lung volume. This not only reflects the severity of lung injury but also, in particular, indicates the limitations of conventional ventilator therapy in which only positive pressure is used.

In this situation, CENPV using the chamber respirator in combination with PEEP and pressure support was able to recruit and maintain lung volume, as indicated by persistently improved oxygenation over five days with PaO_2_/FiO_2_ values above 300 mmHg, associated with an impressive reduction of alveolar infiltrates. These effects may be caused by the different way in which ventilator pressures are applied, resulting in a much more effective means of distending the lungs and maintaining lung volumes, as the negative pressure acts across a broad surface of both the chest wall and abdomen. Therefore, in both surfactant-depleted rabbits and patients with ARDS, at matched end-expiratory and inspiratory lung volumes, applying lower transpulmonary pressures, CENPV presumably also resulted in better oxygenation as compared to CPPV [7, 8].

We decided to apply negative- and positive-pressure ventilation simultaneously, as we have found this combination to be highly effective in ventilating patients with ARDS. During CENPV, regional pleural pressure gradients probably exhibit a substantially different development compared with those in CPPV [7, 8]. Furthermore, transpulmonary pressures (TPP = alveolar pressure minus esophageal pressure), involved in both ventilator modes, should be cumulative when CENPV is combined with CPPV. We speculate that the distribution of ventilation is much more effective with this combination, resulting in alveolar recruitment, reduction of alveolar edema, and stabilization of recruited lung volume, even in severe cases of ARDS; we believe that these effects contributed to the favorable outcome in the present patient.

We observed similar effects in most of our other five patients with severe ARDS who were treated in the chamber respirator in 2002 and also in six additional patients with ARDS who were treated in a tank respirator after completing physiological studies [8]. In these (albeit limited number of) patients, no relevant adverse effects were observed that could be directly related to the combined use of negative- and positive-pressure ventilation. However, especially in patients with severe capillary leakage, external edema increased and decreased again under ambient air. In these latter patients, we limited this therapy to only several hours per day. Particularly in hypovolemic patients, we administered intravenous fluid before starting CENPV to avoid a pronounced decrease in blood pressure associated with low intrathoracic pressures and redistribution of intravascular volume. Finally, of all these patients, 50% survived their stay in the intensive care unit.

In line with this current standard of care, the patient described here was intubated and ventilated with CPPV; we then commenced CENPV as a rescue therapy in late-stage ARDS. Because we did not wish to risk applying CENPV in this patient (or other patients) without a tracheal tube during an early stage of lung injury, CENPV was applied only in intubated patients with severe ARDS. Generally, securing the airway with a tracheal tube is still deemed necessary when a patient with lung injury depends on high ventilatory pressures in conjunction with a high FiO_2_ to maintain gas exchange. As in the presented case, noninvasive positive-pressure ventilation frequently fails in ARDS as a function of its severity and does not appear beneficial in severe ARDS [8]. We believe that CENPV has the potential to reduce this high failure rate, even in severe lung injury. Furthermore, after cessation of CENPV, our patient's breathing pattern with a low breathing frequency and high tidal volumes (making almost sole use of his diaphragm) may also indicate the potential of CENPV to reduce weaning failure. However, due to the paucity of data on this topic, randomized trials comparing noninvasive positive- and negative-pressure ventilation in patients with ARDS are needed to further support this hypothesis.

Devices far less clumsy than our self-made tank respirator or chamber respirator are now available. These include a much more practical Goretex suit that is effectively used with a highly efficient negative-pressure ventilator (Pegaso Vent, Dima S.r.l., Bologna, Italy). This device can be synchronized with spontaneous breathing and can be coupled to a conventional ventilator to optimally synchronize CENPV with CPPV.

Tracheal intubation and CPPV as the current standard of care for ARDS seem to have reached their limit in terms of a further relevant reduction of the still very high mortality rate [2, 3]. This unacceptably high mortality rate calls for alternative techniques that substantially differ from the present methods. CENPV might be worth considering as an alternative, as it has proven less injurious and resulted in better oxygenation in experimental lung injury [7] and also improved gas exchange in a physiologic study on patients with ARDS [8].

The present report is the first to demonstrate how both techniques can be successfully combined in an intubated patient with severe late-stage ARDS. We believe that this therapy made a substantial contribution to the patient's positive outcome and survival. The possibility of effectively applying this and similar concepts, not only in intubated but also in nonintubated patients with ARDS, offers new options that may genuinely differ from the present therapeutic approaches. Therefore, these options may have the potential to decrease the ongoing high mortality rate associated with ARDS.
